# Supplementary material for: BMPs direct sensory interneuron identity in the developing spinal cord using signal-specific not morphogenic activities
Source: eLife. 2017 Sep 19;6:e30647. doi: 10.7554/eLife.30647 (PMC5605194; doi:10.7554/eLife.30647)
Supplement: Supplementary file 1. [file elife-30647-supp1.docx]

**Supplemental File 1:** Antibody information

| **Antigen** | **Species** | **Dilution** | **Source** |
| --- | --- | --- | --- |
| Sox2 | Goat | 1:2000 | Santa Cruz Biotechnology (sc-17320) |
| Pax3 | Goat | 1:500 | R&D Systems (AF2457) |
| Lhx2/9 | Rabbit | 1:1,000 | gift from Tom Jessell; Liem *et al*, Cell, 1997 |
| Mafb | Rabbit | 1:250 | Novus Biologicals (NB600-266) |
| Pax2 | Rabbit | 1:500 | Invitrogen (71-6000) |
| pHistoneH3 | Rabbit | 1:1000 | Cell Signaling Technology (9701) |
| C-terminal phosphorylated Smad1/5/8 | Rabbit | 1:1000 | gift from Ed Laufer; Hazen *et al,* Dev Biology, 2012 |
| GFP | Rabbit | 1:4000 | Invitrogen (A6455) |
| Lhx1/5 | Mouse | 1:20 | Developmental Studies Hybridoma Bank (4F2) |
| Isl1/2 | Mouse | 1:100 | Developmental Studies Hybridoma Bank (39.4D5) |
| p27 | Mouse | 1:500 | BD Biosciences (610241) |
| Tlx3 | Guinea Pig | 1:200 | gift from Thomas Müller; Muller *et al* Genes & Dev, 2005 |
| Bhlhb5 | Guinea Pig | 1:2000 | gift from Bennett Novitch; Skaggs *et al,* Development, 2011 |
| BrdU | Rat | 1:2000 | Harlan Sera-Lab Ltd (MAS250) |

**Additional References (see also reference list)**

Müller T, Anlag K, Wildner H, Britsch S, Treier M, Birchmeier C. 2005. The bHLH factor Olig3 coordinates the specification of dorsal neurons in the spinal cord. Genes & Development 19:733–743.

PubMed: 15769945

DOI: 10.1101/gad.326105
