# Supplementary material for: BMPs direct sensory interneuron identity in the developing spinal cord using signal-specific not morphogenic activities
Source: eLife. 2017 Sep 19;6:e30647. doi: 10.7554/eLife.30647 (PMC5605194; doi:10.7554/eLife.30647)
Supplement: Supplementary file 2. [file elife-30647-supp2.docx]

**Supplemental File 2:** Chicken primer sequences for *in situ* hybridization experiments

| **Gene of Interest** | **Primer Sequences** |
| --- | --- |
| *Bmp4* | F: (with T7 polymerase site) 5’- taa tac gac tca cta tag ggG CTA CCA GGC GTT TTA CTG C -3’  R: (with GAG and T3 polymerase site) 5’- GAG att aac cct cac taa agg gaT TTT CAG CAC CAC CTT GTC A -3’ |
| *Bmp7* | F: with T7 polymerase site) 5’- taa tac gac tca cta tag ggC AAA TGT GCC TCA GTT CCA A -3’  R: (with GAG and T3 polymerase site) 5’- GAG att aac cct cac taa agg gaG CGA GAA AAG CTA ACC CTT G -3’ |
| *Bmp5* | F: with T7 polymerase site) 5’- taa tac gac tca cta tag ggC TTG CTG ATG AGA AGG CAG TT -3’  R: (with GAG and T3 polymerase site) 5’-GAG att aac cct cac taa agg gaG CAG TTA CAG AAG TTC CGG AGT-3’ |
| *Bmp6* | F: (with T7 polymerase site) 5’-taa tac gac tca cta tag ggt aaa atg cga ccc tat gct g-3’  R: (with GAG and T3 polymerase site) 5’-GAG att aac cct cac taa agg gaa ggg acc caa aag att tgc t-3’ |
| *Atoh1* | F: 5’- CAG AGT TTC CGT TGC CCA GA -3’  R: (with GAG and T3 polymerase site) 5’- GAG att aac cct cac taa agg gaT TGG CTG GGC TAA AAT TCA AAT -3’ |
| Ascl1 | in situ probe was made from linearized plasmids, from Gaber *et al*, PLoS Biology, 2013 |
| *Ngn1* | in situ probe was made from linearized plasmids, from Jasoni el at, Development, 1994 |

**Additional references**

Gaber ZB, Butler SJ, Novitch BG. 2013. PLZF regulates fibroblast growth factor responsiveness and maintenance of neural progenitors. PLoS Biology 11:e1001676.

PubMed: 24115909

DOI: 10.1371/journal.pbio.1001676

Jasoni CL, Walker MB, Morris MD, Reh TA. 1994. A chicken achaete-scute homolog (CASH-1) is expressed in a temporally and spatially discrete manner in the developing nervous system. Development 120:769–783.

PubMed: 7600956
