# Supplementary material for: BMPs direct sensory interneuron identity in the developing spinal cord using signal-specific not morphogenic activities
Source: eLife. 2017 Sep 19;6:e30647. doi: 10.7554/eLife.30647 (PMC5605194; doi:10.7554/eLife.30647)
Supplement: Supplementary file 3. [file elife-30647-supp3.docx]

**Supplementary File 3:** Mouse primer sequences for *in situ* hybridization experiments

| **Gene of Interest** | **Primer Sequences** |
| --- | --- |
| *Bmp4* | *F:* (with T7 polymerase site) 5’ -taa tac gac tcc tat agg gGC TAC CAG GCG TTT TAC TGC -3’  *R:* (with GAG and T3 polymerase site) 5’- GAG att aac ccc ata aag gga TTT TCA GCA CCA CCT TGT CA-3’ |
| *Bmp7* | *F:* primer (with T7 polymerase site) 5’- taa tac gac tca cta tag ggg gaa gca tgt aag ggt tcc a -3’  *R:* (with GAG and T3 polymerase site) 5’ -GAG att aac cct cac taa agg gaa ggc ttg cga tta ctc ctc a -3’ |
| *Bmp5* | *F:* (with T7 polymerase site) 5’- taa tac gac tca cta tag ggT GCT GAT GAG AAG GCA GTT C -3’  R: (with GAG and T3 polymerase site) 5’- GAG att aac cct cac taa agg gaG CAG TTA CAG AAG TTC CGG AGT -3’ |
| *Bmp6* | *F:* (with T7 polymerase site) 5’- taa tac gac tca cta tag ggt aaa atg cga ccc tat gct g -3’  *R:* (with GAG and T3 polymerase site) 5’- GAG att aac cct cac taa agg gaa ggg acc caa aag att tgc t -3’ |
