# Supplementary material for: BMPs direct sensory interneuron identity in the developing spinal cord using signal-specific not morphogenic activities
Source: eLife. 2017 Sep 19;6:e30647. doi: 10.7554/eLife.30647 (PMC5605194; doi:10.7554/eLife.30647)
Supplement: Supplementary file 4. [file elife-30647-supp4.docx]

**Supplemental File 4:** Mouse primer sequences for qRT-PCR

| **Gene of Interest** | **Primer Sequence** |
| --- | --- |
| *GapDH* | Primer sequence from Lelievre *et al,* Dev. Bioly, 2008 |
| *Msx1* | F: 5’- gtg cag agg cca aga gat tt -3’  R: 5’-tct ggt ctc ctt cag cct cta -3’ |
| *Lhx2* | F: 5’- cag ctt gcg caa aag acc -3’  R: 5’- taa aag gtt gcg cct gaa ct -3’ |
| *Foxd3* | F: 5’- ccc caa cac tga cca aca g -3’  R: 5’- gtt tgc tcc gcc agc tta -3’ |
| *Isl1* | F: 5’- agg aca aga aac gca gca tc -3’  R: 5’- ttc ctg tca tcc cct gga ta -3’; |
| *Pax2* | F: 5’- AAA CGC GAG GAA GAT GTG TC -3’  R: 5’- AAA GAC TCG ATC CAG AGC TTC C -3’ |

**Additional references:**

Lelievre V, Seksenyan A, Nobuta H, Yong WH, Chhith S, Niewiadomski P, Cohen JR, Dong H, Flores A, Liau LM, Kornblum HI, Scott MP, Waschek JA. 2008. Disruption of the PACAP gene promotes medulloblastoma in ptc1 mutant mice. Developmental Biology 313:359–370.

PubMed: 18036580

DOI: 10.1016/j.ydbio.2007.10.031
