# Supplementary material for: BMPs direct sensory interneuron identity in the developing spinal cord using signal-specific not morphogenic activities
Source: eLife. 2017 Sep 19;6:e30647. doi: 10.7554/eLife.30647 (PMC5605194; doi:10.7554/eLife.30647)
Supplement: Supplementary file 5. [file elife-30647-supp5.docx]

**Supplementary File 5:** BMP concentrations used in these studies

| **Figure** | **Concentration information** |
| --- | --- |
| Figures 2, 5, 6 | BMP plasmids: 500ng/μl |
| Figure 3, 7 | BMP recombinant protein: 10ng/ml |
| Figure 4 | Chicken *in vivo*:  BMP plasmids: 5ng/μl (very low), 25ng/μl (low) 50ng/μl (medium), 500ng/μl (high)  Mouse ESCs *in vitro*:  BMP recombinant proteins: 0.15ng/ml (lowest), 1.5ng/ml, 5ng/ml, 10ng/ml, 20ng/ml (highest) |
| Figure 8 | Chicken *in vivo*:  Dominant Negative BMP receptors: 1μg/μl  BMPs: 50ng/μl (low) 500ng/μl (high)  Mouse ESCs *in vitro*:  BMP recombinant protein: 10ng/ml  BMP receptor inhibitors:  Dorsomorphin: 1x: 1μM, 5x: 5μM, 10x: 10μM  LDN-193189: 1x: 100nM, 10x: 1μM, 20x: 2μM |
